# Hypoglycemic effect of aqueous extract of *Parthenium hysterophorus* L. in normal and alloxan induced diabetic rats

**DOI:** 10.4103/0253-7613.43167

**Published:** 2008-08

**Authors:** Vijay S. Patel, V. Chitra, P. Lakshmi Prasanna, V. Krishnaraju

**Affiliations:** Department of Pharmacology, SRM College of Pharmacy, Kattankulathur-603203, Kanchipuram district, TN, India

**Keywords:** Diabetes mellitus, hypoglycemia, *Parthenium hysterophorus*

## Abstract

**Objectives:**

To study the effects of *Parthenium hysterophorus* L. flower on serum glucose level in normal and alloxan induced diabetic rats.

**Materials and Methods:**

Albino rats were divided into six groups of six animals each, three groups of normal animals receiving different treatments consisting of vehicle, aqueous extract of *Parthenium hysterophorus* L. flower (100 mg/kg) and the standard antidiabetic drug, glibenclamide (0.5 mg/kg). The same treatment was given to the other three groups comprising alloxan induced diabetic animals. Fasting blood glucose level was estimated using the glucose oxidase method in normal and alloxan induced diabetic rats, before and 2 h after the administration of drugs.

**Results:**

*Parthenium hysterophorus* L. showed significant reduction in blood glucose level in the diabetic (*P*<0.01) rats. However, the reduction in blood glucose level with aqueous extract was less than with the standard drug glibenclamide. The extract showed less hypoglycemic effect in fasted normal rats, (*P*<0.05).

**Conclusion:**

The study reveals that the active fraction of *Parthenium hysterophorus* L. flower extract is very promising for developing standardized phytomedicine for diabetes mellitus.

## Introduction

Diabetes mellitus is a chronic metabolic disorder affecting approximately 1.5% of the total population that continues to present a major worldwide health problem. It is characterized by absolute or relative deficiencies in insulin secretion and/or insulin action associated with chronic hyperglycemia and disturbances of carbohydrate, lipid and protein metabolism. As a consequence of metabolic derangement in diabetes, various complications develop, including both macro and micro vascular dysfunctions,[1] where complete cure with insulin and oral hypoglycemic agents without side effect has been challenging.[2,3] Many herbal products, several metals and minerals have been prescribed for the cure of diabetes mellitus alone or in combination with oral hypoglycemic agents, in ancient literature.[4,5] *Parthenium hysterophorus* L., compositae, also known as congress weed, carrot weed, star weed, white top, chatak chandani, bitter weed, ramphool, and gajar grass, reaching a height of 2 m in good weeks of germination, is believed to have entered India accidentally in the mid 1950's and is now available abundantly all over the India. All parts of the plant are reported to be used as bitter tonic, febrifuge, emmenagogue, antidyscentric etc.[6] The plant is used by some people in the state of Maharashtra and Gujarat (India) in the treatment of diabetes mellitus. However, no scientific data is available regarding the effect of *Parthenium hysterophorus* L. on blood glucose level. The present study was undertaken to explore the effect of the flower extract of *Parthenium hysterophorus* L. on the blood glucose level of experimental animals.

## Materials and Methods

Fresh plant of *Parthenium hysterophorus* L. was collected in the month of July-August and was authenticated at the Plant Anatomy Research Center, Chennai. The flowers were dried under sunlight and powdered. 20 gm of the powdered drug was boiled with 100 ml of distilled water for four hours.[7] To obtain water extract, the residue was removed by filtration and the filtrate was evaporated to dryness. The yield of extract was about 2.6 gm from 20 gm of dried flower powder. The extract was suspended in 5% Tween 80 and used for oral administration.

### Animals

Adult albino wistar rats, of either sex, weighing 150-200 g, were acclimatized for a period of 10 days at room temperature and 50% relative humidity. They were housed in a standard cage and maintained on standard pellets and water at libitum. The animals described as ‘fasted’ were deprived of food for 18 h, but had free access to water.

### Experimental setup

The fasted rats were divided into six groups of six animals each (three groups of normal animals and three groups for induction of diabetes). The normal animals were given the following drug treatment after 18 h of fasting. The diabetic animals were given the same treatment after 72 h of alloxan administration.

**Table:** 

**Group A:** (Control):	Received 0.5% Tween 80.
**Group B:** (Positive control):	Received aqueous suspension of glibenclamide 0.5mg/kg in 0.5 ml of 5% Tween 80.
**Group C:** (Test group):	Received aqueous extract of *Parthenium hysterophorus* L. 100 mg/kg with 0.5 ml of 5% Tween 80.

All the drugs were administered in a single dose, with the help of a stomach tube. The dose of the standard drug glibenclamide was calculated on the basis of human dose, based on surface area by extrapolation method.[8] The institutional ethics committee approved all experimental protocol.

### Hypoglycemic study in normal rats

The fasting blood glucose level was monitored in blood sample collected from the ear vein, using the glucose oxidase method.[9–10] The blood glucose level of the different groups was estimated 2 h after the administration of the drug. The period of 2 h is based on the finding that the maximum hypoglycemic effect of glibenclamide was found around two hours of administration.

### Hypoglycemic study in Alloxan induced diabetic rat

Alloxan monohydrate (150 mg/kg body weight) dissolved in normal saline and injected i.p. in 18 h previously fasted animal to induce diabetes. After one hour of alloxan administration, the animals were fed standard pellets and water at libitum.[11] After 72 h, the blood glucose levels were estimated, applying the glucose oxidase method and rats having blood glucose level more than 150 mg/dl were selected for the study. Fasting blood glucose level before and 2 h after the administration of the drug were estimated.

### Estimation of blood glucose

Blood glucose was estimated by autoanalyser using a commercial assay kit (ERBA diagnostics mannchim GmbH, Germany). The blood sample was centrifuged at 3000 rpm for 20 min and 10 *µ*l serum was used for each assay.[12]

## Results

In alloxan induced diabetic rats, those animals with blood glucose level in the range of 280-310 mg/dl were considered as severe diabetics. All animals survived without any side effect and mortality. In the test group, the blood glucose level significantly (P< 0.01) reduced below 240 mg/dl (21%) at 2 h while in the glibenclamide treated group, the blood glucose level reduced to 30%. In the normal rat, the percent decrease in blood glucose level at 2 h with glibenclamide was 10%, while it was only 6% with *Parthenium hysterophorus* L. extract [Table 1]. The aqueous extract of *Parthenium hysterophorus* L. significantly (P< 0.01) decreased fasting blood glucose level in alloxan induced diabetic rat at 2 h. However, reduction in blood glucose level is less than glibenclamide treated group.

**Table 1:** Effect of aqueous extract of *Parthenium hysterophorus* L., on blood glucose level of normal and alloxan induced diabetic rats (n=6). Values are mean ± SEM

*Groups*	*Treatment*	*Dose and route*	*Blood glucose level, (mg/dl)*					
			
				*Normal group*			*Diabetic group*			
				
			*Pretreatment*	*Posttreatment*	*% Decrease*	*Pretreatment*	*Posttreatment*	*% Decrease*
Control	Tween 80	0.5ml/kg (oral)	79.20±0.35	77.01±0.32	2.76	297.53±3.62	296.74±2.96	0.3
Positive control	Glibenclamide	0.5mg/kg (oral)	79.58±0.37	71.22**±0.70	10.50	301 21±1.02	210.73**±3.07	30.03
Test	*Parthenium hysterophorus* L.	100mg/kg (oral)	80.02±0.49	74.98*±0.63	6.29	299.20±2.02	236.34**±2.21	21.00
One-way ANOVA		F	0.98	26.03		0.89	279.99	
		df	2, 15	2, 15		2, 15	2, 15	
		P	0.746	0.280		0.041	0.816	

* *P*<0.05

** *P*<0.01 as compare to the control

## Discussion

The study reports the hypoglycemic activity of aqueous extract of *Parthenium hysterophorus* L. If the active principle(s) is/are identified, it can lead to the development of a potent allopathic medicine. Further, it is interesting to note that the drug exhibited significant hypoglycemic activity only in alloxan induced diabetic animal, as compared to its effect in normal animals. Alloxan induces diabetes by destroying β-cells of pancreas, through production of reactive oxygen species.[13] Therefore, unlike the clinically used oral sulphonylurease, this herbal drug does not seem to work by stimulating β-cells and releasing insulin. This suggests that its main mechanism of action may not be potentiation of insulin release from pancreatic β-cells and therefore the drug could be effective in insulin independent, type II diabetes mellitus also.

The present study demonstrates that aqueous extract of Parthenium hysterophorus decrease glucose level in alloxan induced diabetic animal. Further studies are in progress in the laboratory to elucidate in detail the actual mechanism of the action of this drug at the cellular and molecular levels. 
